# Socially Anxious and Confident Men Interact with a Forward Virtual Woman: An Experimental Study

**DOI:** 10.1371/journal.pone.0032931

**Published:** 2012-04-11

**Authors:** Xueni Pan, Marco Gillies, Chris Barker, David M. Clark, Mel Slater

**Affiliations:** 1 Department of Computer Science, University College London, London, United Kingdom; 2 Department of Computing, Goldsmiths, University of London, New Cross, London, United Kingdom; 3 Department of Clinical, Educational and Health Psychology, University College London, London, United Kingdom; 4 Department of Experimental Psychology, University of Oxford, Oxford, United Kingdom; 5 ICREA-Universitat de Barcelona, EVENT Lab, Facultat de Psicologia, Barcelona, Spain; Barrow Neurological Institute, United States of America

## Abstract

**Background:**

Male volunteers entered an immersive virtual reality that depicted a party, where they were approached by a lone virtual woman who initiated a conversation. The goal was to study how socially anxious and socially confident men would react to this event. Interest focused on whether the socially anxious participants would exhibit sustained anxiety during the conversation or whether this would diminish over time, and differ from the responses of the more socially confident men.

**Methodology:**

The scenario was a party with five virtual characters, four sitting at a distance from the participant and talking amongst themselves and one lone woman standing closer. The woman approached the participant, introduced herself and initiated a conversation that was first about mundane matters and then became more personal and intimate. Participants were men who were either relatively socially confident (18) or socially anxious in their relationships with women (18). A second experimental factor was whether or not the other four characters occasionally looked towards the participant. There was a post-trial questionnaire about social anxiety in relation to the experience, and skin conductance and ECG physiological measures were recorded. Our expectation was that the socially anxious participants would show greater anxiety throughout.

**Conclusions:**

Compared to baseline readings both socially confident and socially anxious groups on average showed signs of significantly increased stress at the initial approach of the virtual woman. The stress then diminished once the conversation entered into the mundane phase and then did not significantly change. Comparing pre- and post-questionnaire anxiety scores there was no change for the more confident participants but a significant decrease in average score amongst the anxious group. The methodology of placing socially anxious participants in a virtual reality where they can gain experience of how to act in a stressful situation promises a novel way forward for treating social anxiety.

## Introduction

One of the most striking types of experience in an immersive virtual reality (IVR) system is one that involves personal interaction with virtual characters. Such characters, although they do not typically look real nor behave as realistic humans, nevertheless are life-sized, can look the human participant in the eye, can make facial expressions, talk, make bodily gestures, and carry out activity in relation to the participant that appears to endow them with life. The focus of the research described in this paper is on a one-to-one interaction between a person and a virtual character, in an apparently free flowing conversation. The specific context is that of using virtual reality to eventually help sufferers from social anxiety to overcome their fears, by demonstrating that people who experience social anxiety in real life, also experience this when they engage with virtual characters, and that therefore this technique can be used in the context of psychological therapy. The extent to which people respond realistically to situations and events within IVR is referred to as ‘presence’ [1].

A reason for the general importance of presence is its practical utility. If virtual characters can evoke a realistic response then they can be used in a variety of settings where the utilisation of real people is either too costly, impractical, or unethical. For example, using such a virtual reality system it was possible to produce a simulation of one of the most controversial experiments in social psychology, the Stanley Milgram obedience experiment [2]. Such experiments, although potentially powerful in throwing light on an important area of human behaviour, have been banned for 50 years due to ethical considerations, although a partial replication of the experiment has been performed with real people [3]. In virtual reality we were able to simulate one of Milgram's conditions by replacing the live human actor receiving the electric shocks as the Learner, by a virtual character. Since all experimental participants knew that in fact they were giving ‘electric shocks’ only to a virtual character, the ethics concern regarding deception was eliminated, and yet the participants still tended to have stress responses indicating that to some extent they were responding to the situation as if real.

In the research described in this paper we were interested in knowing how men who normally experience anxiety in social relationships with women would react to a positive encounter initiated by the woman, and the progress of the anxiety over time. IVR systems have been used in the treatment of anxiety disorders over several years, since they offer a highly flexible method for creating situations that may be useful in the treatment of a range of such disorders, where the corresponding experience is repeatable, without the inconvenience, cost, and possibly danger of creating situations in physical reality. Here we provide a brief review of this literature, followed by the specific application to a situation involving social anxiety.

One of the first applications of IVR in the treatment of anxiety disorders was acrophobia (fear of heights). For example it was reported in [4] that participants exhibited acrophobia signs and symptoms within virtual environment exposures that included an elevator ride, a bridge and a view from a tall building. In [5] this was used for exposure therapy and it was found that compared to a waiting list group the treatment group showed significant reductions in anxiety over an eight week period. In [6] there was comparison of results between an *in vivo* treatment group with a virtual reality treatment group, where the virtual reality depicted the same place as the real place used *in vivo*. It was found that both virtual reality and *in vivo* groups showed similar levels of improvement compared to a control group over a 6 month follow-up period, suggesting that VR was just as good as real experiences in the context of exposure therapy. There have been other studies reporting successful results – for fear of flying [7], [8], arachnophobia [9], [10], claustrophobia [11], [12], and agoraphobia [13]. Overall a meta-analysis of 13 studies of virtual reality exposure therapy found that this was at least as effective as in vivo treatments that did not employ virtual reality [14].

The use of virtual environments for the treatment of socially related conditions is far more challenging than the types of application considered above, and is not commonly tried. Indeed in the meta-analysis in [14] only two out of the thirteen studies were concerned with social phobia. In a follow up investigation [15] the application of virtual reality exposure therapy was considered in 20 studies, only two of which concerned social phobia, and one of these was concerned with the more specific problem of fear of public speaking. The reason for this relative lack of attention to social anxiety is clear. Consider, for example, fear of heights – here it is enough for the virtual environment to depict a static scenario (showing some sort of precipice) and the main interaction by the patient within this environment is to move and look around. For social disorders, however, there is a far greater computational problem, which is the depiction in virtual environments of virtual humans realistic enough that the appropriate affect is generated. How realistic must these virtual humans be? Do they have to be like real people for this to work at all, or can relatively crude representations be used to achieve realistic results? The answer appears to be that representations that have sufficient behavioural fidelity, and enough visual fidelity so as to be clearly recognised being like humans will evoke anxiety in socially anxious people (for example, [16], [17]). In the Milgram experiment and in the experiment reported in this paper, the level of realism of the virtual humans, both with respect to absolute appearance and realism of body movement was only moderate.

The type of social anxiety that has attracted most attention with VR therapy has been fear of public speaking [16], [17], [18], [19], [20], [21], [22], [23]. It was shown in [19] that using a virtual environment that depicted a static audience within an auditorium may have been sufficient to improve the condition of a treatment group in exposure therapy compared to a control group. In [16] people were exposed to different types of dynamically responding audiences in an IVR. Those exposed to a positive audience experienced positive emotional responses and those exposed to a negative audience exhibited strong signs of anxiety. It was later shown that the presence of the audience itself makes a difference – people with strong fear of public speaking showed significantly greater anxiety on a range of measures in response to a virtual environment depicting a virtual neutral audience compared to a virtual environment depicting an empty room [17], whereas there was no difference in anxiety between these two conditions for a group of confident public speakers. These results may appear to be obvious (that people respond with negative affect to negatively behaving audiences, or that people with fear of public speaking are more anxious in a room with an audience compared to an empty room) but it has to be remembered that in all cases there was no audience there, only virtual characters within a virtual reality.

The use of IVR in more generalised social situations – such as attendance at meetings, parties, dances, restaurants, and so on – has received less attention. Fear of public speaking, although involving virtual people, nevertheless is still largely a one-way flow – one person talks (the real person) and (virtual) others (appear to) listen. In a more generalised situation the flow is two-way, people interact with each other, a virtual character can approach the participant, and engage in a conversation. This is computationally far more difficult to achieve. There is a slightly more generalised scenario described in [24] depicting some office situations where the participants were required to speak. In order to avoid the problem of programming the virtual characters to exhibit highly realistic behaviours and facial expressions, they concentrated largely on correctly rendering the eyes of the virtual characters in order to ensure that the participants were always being observed. Another similar study was carried out by members of the same group [25] who although concentrating on speaking in public, generalised the situations somewhat. Another interesting approach in that paper was to measure eye contact avoidance as a means of assessing the degree of engendered anxiety. There was a further generalisation achieved in [26] who describe a VR protocol for four different situations: performance (speaking in front of others), intimacy (establishing contacts, introducing oneself), scrutiny (carrying out tasks while others are looking), assertiveness (responding to criticism, responding to people blocking a doorway, and so on). A very similar study is described in [27]. The studies, described as preliminary, were carried out on 10 and 36 subjects respectively, and with a waiting list group, with a CBT treatment group and a VR treatment group. The virtual reality display was just a 2D screen, and the characters were videos of real people. The problem with the former is that immersion is low, and probably also the resulting presence. More importantly, as with so many of the studies described here, there was still no adequate demonstration that the virtual reality or the virtual characters portrayed were having any specific effect. The problem is that doing *anything* may also have the same effect, or displaying a virtual reality without any social content may also have the same effect. This is unlike the study in [17] where it was shown that the anxiety response to the virtual environment that contained people was significantly higher than the one that contained the empty room, for the socially anxious participants.

More generalised situations have also been tackled. In the study described in [28] the participants entered a virtual party, and the several characters there spoke to and waited for answers from the participants. This did not require any sophisticated speech recognition or construction of arbitrary sentences by the virtual characters, rather off-line recordings of a number of phrases were made, and these were triggered appropriately by an unseen operator controlling the operation of the computer program, as in the experiment described in this paper. Other social situations have been successfully portrayed in full immersive VR, again depicting a party situation where several characters interact with the participant [29]. Physiological recordings showed that people were aroused when the virtual characters spoke to them and in particular people with high scores on a social anxiety scale also experienced stress (as measured by ECG analysis).

The experiment that we describe in this paper is unique in the type of social encounter that is depicted – which should normally be experienced as a pleasant one were it to occur in everyday reality. Results of earlier pilot experiments that used a similar set up, and which led to the current study can be found in [30], [31]. Here an attractive woman initiates contact with the male participant – with the conversation becoming increasingly personal over time. Previous work concentrating on social anxiety has been largely non-interactive in the sense that there was no two-way flow of conversation between people and virtual humans – rather there are specific highly constrained encounters such as in public speaking, or particular situations with participants having to carry out various tasks [27]. Our focus was on social anxiety amongst males in relation to an apparently contingent and open-ended social encounter with a female, moreover one who takes the lead in initiating the conversation. For shy males such encounters with females normally do not occur because they avoid situations (such as parties, dances, etc) due to the foreseen stress. This is part of their strategy of engaging in safety behaviours that are designed to avoid the anxiety provoking situation [32]. Our hypothesis was that shy males would show considerable anxiety when put into a situation of such a social encounter, whereas socially confident males would not. However, we did not know whether a short time exposure of shy males to such an encounter would lead to adaption with their anxiety lessening during the course of the encounter.

## Methods

### Ethics Statement

The experiment was approved by the UCL Research Ethics Committee, and was carried out under written informed consent from each participant.

### Materials

The experiment was conducted in a Cave-like projection based system [33] – a Trimension ReaCTor. This has three back-projected vertical screens (front, left and right) (3 m×2.2 m) and a floor screen (from a ceiling mounted projector) (3 m×3 m) controlled by a Silicon Graphics Onyx 2. Head tracking was performed with an Intersense IS 900. The participants were fitted with a Mindmedia Nexus 4 physiological recording device that recorded ECG (1024 Hz) and skin conductance (128 Hz). Electrodes were placed on the palmar areas of the index and ring fingers of the non-dominate hand in order to record electrodermal activity. Electrodes were placed on the left and right collarbones and the lowest left rib in order to record ECG.

### The Virtual Environment

**Figure 1:**
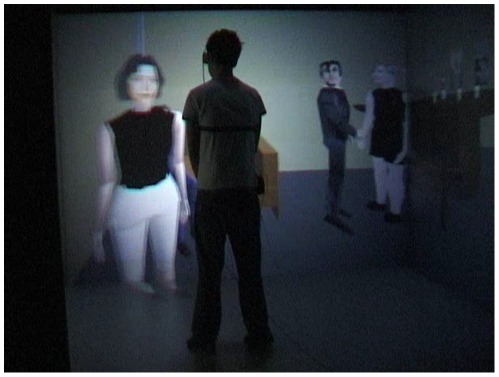
A Photograph from Outside the Cave. A photograph from outside of the Cave as the Christina avatar begins to approach the participant. Other characters can be seen in the background. The image is blurry since the Cave projections are double imaged due to the stereo.

The virtual environment in which the experiment took place was a room populated by 5 virtual characters (avatars) 4 of which were talking to one another, except for one lone female virtual character (‘Christina’) (Figure 1). There was background music playing as if in a party. Once the participant entered the scenario the Christina avatar gazed towards him for a few seconds, then made her way towards him, and initiated a conversation. It is important to note that from the point of view of the participant the Christina avatar was life-sized (as shown in Figure 1) and that the projection was active stereo. The participant stood approximately in the centre of the room and head tracking was used so that the Christina avatar was programmed to maintain a social distance of 1 m and look towards the participant's face, and also the other characters in the room might occasionally look towards him.

### The Christina Avatar

Christina was modelled to be an attractive female, speaking English with a slight Portuguese accent (the accent of the real actor who recorded the script). She started the conversation with the participant by introducing herself and asking the participant questions. When he spoke, she appeared to listen carefully and showed her interest by nodding and smiling. She also showed her interest by leaning forward to him, smiling while looking at him, and also by eventually breaking the norms of social distance by approaching within 0.5 m. Finally she suggested they should meet up again. The whole conversation lasted about 3 minutes.

Christina's behaviour consisted of a number of multi-modal utterances, containing recorded speech, body and facial animation, which were triggered from a control panel by an experimenter, who was listening to but out of view of the participant. There were around 60 utterances prepared and pre-recorded; half of these were the core of the conversation and the rest were backups for contingent situations. Each utterance was a synchronised combination of speech (audio file) and movements (animation). The animation engine distinguished between foreground behaviour, the multi-modal utterances, and background behaviour including gaze and proxemics. We used a model of proxemics that ensured that Christina mostly oriented towards the participant and maintained an appropriate conversational distance, but later breaking this as she approached closer to the participant.

### The Conversation

The following is an extract from a typical conversation between the Christina Avatar and a participant: (Christina – C; Participant – P). An example of a complete dialog can be found in Text S1 and in Video S1.

C: This shirt looks great on you, how much was it?

P: Thank you! It is a gift.

C: Ah, I really want to find a pair of trousers something like those for my brother [glancing down at the man's trousers]. Where did you get those?

P: Haha, I cannot remember, but there are a lot of nice shops along Oxford Street if you are interested.

C: [Turns her face to the other virtual characters around] So, Do you know anyone here?

P: Well, not really anyone else, no.

C: I feel a bit shy about talking with the other people, do you mind if I talk with you for a bit longer?

P: Sure, no problem.

### Factorial Design

The experiment was a 2×2 between-groups factorial design, with factors of Social Anxiety and Others' Gaze. The first factor (Anxiety) was whether the participant normally experiences social anxiety in relationship with members of the opposite sex or whether he is socially confident in this regard. This was determined by a subset of the SPAI (Social Phobia and Anxiety Inventory) questionnaire [34]. The full SPAI questionnaire has 45 items consisting of a total of 110 questions. Thirty two of the items (consisting of 97 questions) are concerned with social phobia and the remaining 13 items are concerned with agoraphobia. Each question is scored on a scale ranging from 1 to 7 where higher scores indicate greater anxiety. For example, “I feel anxious in small gatherings with strangers" where “Never" is scored as 1 and “Always" as 7. The original idea of the SPAI questionnaire was to compute a total score based on the social phobia items and subtract the total score based on the agoraphobia items – thus ending up with a score that reflects a pure social phobia scale, that is social phobia that is not itself a secondary symptom of agoraphobia.

In this experiment we were not at all concerned with full social phobia or clinical assessment of that condition. Instead the SPAI questionnaire was used as a source of questions related to the main issue of interest – males who were shy in their relations with females, and associated resulting behaviours such as feeling anxious in social situations where women might be present. For the selection of participants we used 8 SPAI questions related to the experimental scenario concerned with relations with members of the opposite sex and an additional question of special relevance (“I feel anxious when being approached by opposite sex"). Altogether we used 32 questions from the SPAI, plus one additional set of 4 questions “I feel anxious when being approached by …[strangers, authority figures, opposite sex, people in general]". We wanted to avoid the participant realizing that we were mainly interested in encounters with the opposite gender. We call this the *pre-Exposure* questionnaire. The questions selected are shown in Table S1.

Participants were recruited by posters and email on the campus at University College London to all levels of staff and students. People interested in participating in the experiment were invited to complete the pre-Exposure questionnaire online. The questionnaires were then processed based on the 9 questions about anxiety in dealing with the opposite sex. The number of respondents was 153, and their mean questionnaire scores on the 9 questions were ranked from lowest to highest. Our final sample was recruited by working upwards from the lowest level (more confident) scores and working downwards from the highest level (more anxious) scores until we had recruited 18 in each group. The resulting Confident group had the range of pre-Exposure scores (restricted to questions about relations with the opposite sex) between 1.22 and 2.44 (median=1.95) and the Anxious group between 4.00 and 6.33 (median=5.06) where the maximum possible score was 7. For the full set of 36 questions the equivalent values are 1.33 to 2.67 (median=1.93) for the Confident group and 3.25 to 6.00 (median=4.58) for the Anxious group. There was therefore a clear separation between the two groups.

The second experimental factor (Others) was the extent to which the other virtual characters in the bar occasionally looked towards the participant. In one condition once the conversation had started the other characters did not look towards the participant (Not-Observed), and in another condition they did look frequently (Observed). Our interest was to discover whether social anxiety would be greater for participants in the Observed condition [32]. The finally achieved design is shown in Table 1. The Others factor is unbalanced due to some low quality ECG physiological data recordings in the original sample (the SPAI ranges above are for the actual final sample used as in Table 1).

**Table 1:** Factorial Design – The Final Number of Participants Distributed by Factor.

	Others	
Anxiety	Not-Observed	Observed
Confident	9	9
Anxious	10	8

The mean age of the 36 participants was 26 (range from 18 to 35) with no significant difference in age between the 4 cells. Six were undergraduates, 14 Master students and 9 PhD students. The rest were 1 university staff, 1 architect, and 1 IT worker. The other 4 did not specify their occupation.

### Procedures

After informed consent procedures, each participant was asked to complete a pre-questionnaire giving basic information such as age, occupation, etc. Then he was introduced into the Cave and fitted with shutter glasses, a head tracker and the Nexus physiological recording device, and a microphone in order for the experimenter to hear the conversation. This was then followed by the actual experiment. Then the participant completed a post-Exposure questionnaire (see later section) and a presence questionnaire (e.g., [1]). Finally a short interview was held. The whole procedure took between 45 and 60 minutes. The experimental operator (female) and an assistant (male) were present throughout the whole experiment. However, they could not be seen while the participant was experiencing the environment in the Cave.

### Phases of the Experiment

There were four distinct phases to the experiment: (1) *baseline* – where the participant was in the displayed bar alone, music was playing but nothing else happened; (2) *initiation* – where the characters were all present and the virtual woman approached the participant and initiated a conversation; (3) *mundane* – where the conversation was mainly about everyday matters such as places for living and work; and (4) *intimate* – where the character moved to intimate distance, personal matters were discussed, compliments given, and the issue of another meeting raised. The detailed interaction including all the phrases uttered by the character is shown in Table 2.

**Table 2:** Phases of the Experiment.

Baseline		Time (s)
1	*Baseline starts*	
2	*Baseline ends*	150
**Start**: The virtual female initiates the conversation.		
3	*Experiment starts*	
4	*Avatar stares at the participant*	
5	*Avatar approaches to within normal social distance*	
6	“Hi, It looks like we are the only people alone here, right?"	
7	“My name is Christina."	
8	“It's very nice to meet you."	
9	“So, what are you doing for a living?"	
10	“Very interesting, tell me more."	
11	“I'm an air hostess; I just arrived in London yesterday. Where do you live?"	50
**Mundane**: the conversation is about mundane matters		
12	“I don't know London very well, but actually, I am thinking about moving here, what do you think?"	
13	“But I heard it rains all the time here, is that true?"	
14	“Well, the weather is not that important to me. Have you lived here long?"	
15	“Do you like it here?"	
16	“I've noticed that people dressed very well around here. By the way, that shirt looks great on you. How much was it?"	
17	“Ah, I really want to find a pair of trousers, something like these (*Looking down*) for my brother. Where did you get these?"	50
**Intimate**: the conversation became more intimate		
18	“So, do you know anyone here?"	
19	“I feel a bit shy about talking with other people, do you mind if I talk with you for a bit longer?"	
20	*The avatar approaches to an intimate distance*	
21	“If you don't mind me saying, I think you look very nice."	
22	“I was wondering actually, are you single, or involved with someone at this time?"	
23	“Actually, it is the same with me."	
24	“Anyway, what are you doing after this party?"	
25	“Maybe we should meet up."	50

The times shown are the total for the phase. For Baseline this is accurate but the times shown for the others do not include the varying amount of talking time of the participants.

### Questionnaire Measures for Anxiety

A post-Exposure questionnaire was administered immediately after participants left the virtual environment. This was an alternate form of the SPAI questionnaire consisting of a subset of 35 items. In order for these items to make sense for the participants they were slightly edited from the original wording by tense changes, so that they referred to the specific experience that the participants had just had rather than to life in general (Table S1). For instance, the item from the SPAI “I feel anxious and I do not know what to do in a new situation with members of the opposite sex", was adjusted in the post-Exposure questionnaire to “I felt anxious and did not know what to do in this new situation with members of opposite sex". We specifically wanted the second questionnaire to be about the actual situation that had just been experienced rather than life in general. The interest was in how their responses to this particular scenario might be different to their experiences in everyday life.

Since both pre- and post-Exposure questionnaires are subsets of the full SPAI (albeit with some slight changes regarding tense in post-Exposure) we should expect that they would positively correlate with the full SPAI and thus function as alternate forms of the scale. There is some evidence for this since, for example, it was shown in [35] that individual items are generally positively correlated with the overall score. Indeed in the construction of the SPAI questionnaire items that did not correlate strongly with the total score were eliminated [34]. It was also shown that a smaller sized questionnaire could be constructed that correlates well with the full 32-item questionnaire [35]. In Text S2 we also show that there is likely to be a strong positive correlation between the total score SPAI score and the total score on a subset questionnaire.

One test of the consistency between the pre- and post-Exposure is with respect to those in the Confident group. Here we would expect high consistency between the pre- and post- scores, since the experimental manipulation was such that this group should have been unaffected by it with respect to their self-reported level of anxiety (they were specifically selected on confidence in their relationship with members of the opposite sex). Hence the post-Exposure scores for this group should be predictable from their pre-Exposure scores. In other words the correlation between pre- and post-Exposure scores should be high, the regression line should have slope not significantly different from 1 and with intercept not significantly different from 0. Indeed this is the case. The Pearson correlation is r=0.57 with regression slope 0.96 (P=0.014) and the intercept is 0.24, not significantly different from 0 (P>0.73). Hence for the Confident group the relationship between pre- and post-Exposure scores provides some validity for the relationship between the questionnaires. In contrast, for the Anxious group the correlation is not significantly different from 0 (r=0.34, P>0.17) indicating that this group responded differently to the post-Exposure questionnaire compared to the pre-Exposure questionnaire,

### Questionnaire Measures for Presence

Although this experiment was not about presence some questions were included in a questionnaire administered after the anxiety questionnaire. The questions are shown in Table 3, and are based on the idea that realistic responses of participants with respect to virtual situations and events signify presence. ‘Response’ refers to many dimensions ranging from unconscious physiological responses, automatic behavioural responses, conscious behavioural responses, emotional responses, and thoughts [1]. The questionnaire asks about two different aspects of the scenario: about the party and about the virtual woman. The questions asked participants to compare their responses to what these would have been in a similar real situation – with respect to their overall behaviour, what they said, their emotional responses, their thoughts, and physical responses.

**Table 3:** Results of Post Experiment Questions About Responses.

Question	Median	IQR	P (median=4)
**About the party**			
How much was your overall behavior like being in a party?	5	2.5	0.029
How often did you find yourself automatically behaving within the party as if it were a real place?	5	2.5	0.061
How much was what you said like what you would have said in a real situation?	5	2	0.003
How much was your emotional response in the party the same as if it had been real?	4.5	3	0.597
How much were the thoughts you had within the party the same as if it had been a real situation?	5	2.5	0.087
How much were you thinking things like ‘I know this isn't real’ but then surprisingly finding yourself behaving as if it was real?	5	2	0.002
To what extent were your physical responses within the party (e.g., heart rate, blushing, sweating, etc.) the same as if it had been a real situation?	5	1.5	0.002
**About the woman**			
How much did you behave as if the woman were real?	5	3	0.150
How much was what you said to her like what you would have said to a real woman in a similar situation?	5	2.5	0.043
How much did you find yourself automatically behaving as if the woman were real?	5	2.5	0.009
How much was your overall emotional response to the woman as if she were real?	4.5	3	0.473
How much were the types of things you were thinking while talking with her similar to a real situation?	4	3	0.711
How much did you have physical responses (such as change in heart rate, blushing, sweating, etc.) to the woman as if she were real?	4	2	1.000
How much were you thinking things like ‘I know this person isn't real’ but then surprisingly finding yourself behaving as if she was real?	5	2	0.001

Each question was scored on the scale from 1 ‘not at all’ to 7 ‘very much’. In each case the median, interquartile range, and the significance level of the test that the median is 4 is given based on the two-sided sign test.

It should be noted that this is not a validated questionnaire and we make no claim for it as a general measure for presence. We consider it as a useful tool together with other data, providing help towards understanding of how people behaved in the virtual environment in this particular experiment. A similar strategy was used in [36]. It is important to note that we did not expect that there would be any difference between the Confident and Anxious groups since they used the same system and were subject to the identical scenario. This point is taken up further in the Discussion.

### Physiological recordings

The physiological measures recorded during this experiment were electrodermal activity (EDA) [37], and the electrocardiogram (ECG). The participants were fitted with a Nexus 4 physiological recording device that recorded the ECG (1024 Hz) and skin conductance (128 Hz). Electrodes were placed on the palmar areas of the index and ring fingers of the non-dominant hand in order to record electrodermal activity. Electrodes were placed on the left and right collarbones and the lowest left rib in order to record ECG.

EDA measures changes in arousal through changes in skin conductance caused by sweat levels. An important derived measure of interest is the number of Skin Conductance Responses (SCR) which reflect transient sympathetic arousal, either spontaneous or in response to events, specifically the orienting response, that is responses to changes in the environment and events or surprises. Skin conductance responses (SCR) were defined to be local maxima that had amplitude of at least 0.1 µS and in a period not exceeding 5 s from the start of the SCR to its maximal point. (There is no standard definition, and we used these criteria as being a compromise amongst several in the literature). The *amplitude* refers to the maximum level reached compared to the start of the SCR. Of interest are both the number and amplitude of SCRs, and we also refer to the *SCR rate* as the number of SCRs per second.

The ECG was acquired with the Nexus 4 device (sampling frequency: 1024 Hz), and the analysis was performed with the g.BSanalyze biosignal analysis software package (g.tec – Guger Technologies OEG, Graz, Austria). The first step in ECG analysis was to detect QRS (ventricular contraction) complexes in the ECG time series. The QRS complexes determine the distance in time from one heart contraction to the next one (RR interval) and were detected automatically based on a modified Pan-Tompkins algorithm [38]. Then a visual inspection of the detected QRS complexes was performed to correct any missed or wrongly assigned points. The QRS complexes determine the time between heart contractions – the RR intervals. An NN interval refers to normal-to-normal intervals, where non-normal beats such as extra systoles are not taken into account. From the ECG signal and QRS complexes a number of parameters can be derived. In our analysis we have used the following time domain measures (as in [29]): HR – heart rate in beats per minute (bpm), NN50 – number of intervals of successive NN intervals greater than 50 ms [39], and PNN50 [40] the ratio of NN50 to the total NN count. Low PNN50 is typically associated with greater mental stress, for example [41].

## Results

### Being Observed By Others

One of the factors in the experiment was whether or not other virtual characters were sometimes looking towards the participant or not. This factor was not significant in any of the analyses described in subsequent sections, probably because not all those in the Observed group noticed that they were being observed. In response to the question “How much were you aware of the other people in the party looking at you?" on a 1–7 scale where 1 meant ‘not at all’ and 7 ‘very much’, the median scores were 1 for the Non-Observed group and 3 for the Observed group (the difference is significant, P<0.0003, Wilcoxon rank-sum test). In response to the question “To what extent did it disturb you, if at all, if the other people in the party looked at you?" the median scores in the Non-Observed and Observed group were also 1 and 3 respectively (P<0.01) and the median scores for those in the Confident and Anxious groups were 1 and 2.5 respectively (P<0.03). Although non-parametric statistics are preferred in the context of single-item questionnaire responses, we note that a two-way ANOVA with response variable as this ‘disturb’ question and factors as the anxiety level (Confident, Anxious) and whether or not the participant was observed, yields significant differences in the means (anxiety, P<0.005 and observed, P<0.002) with no interaction effect. (The response variable was on a log scale in order to obtain normally distributed residual errors).

### Pre- and Post-Exposure Questionnaires

Here we consider the difference in scores between the post-Exposure and the pre-Exposure questionnaires. For the Confident group the median of the differences is 0.15 with an interquartile range of 0.64. Using the sign test this is not significantly different from 0 (P>0.48, two-sided). For the Anxious group the median is −1.00 with interquartile range 1.14, which is significantly different from 0 (P<0.008, two-sided). These two medians are significantly different (P=0.0003, two-sided, Mann-Whitney U-test). There was therefore no difference in score between the pre- and post-Exposure questionnaires for the Confident group, but for the Anxious group the post-Exposure questionnaire had on the average a significant reduction of 1 point.

### Skin Conductance

**Figure 2:**
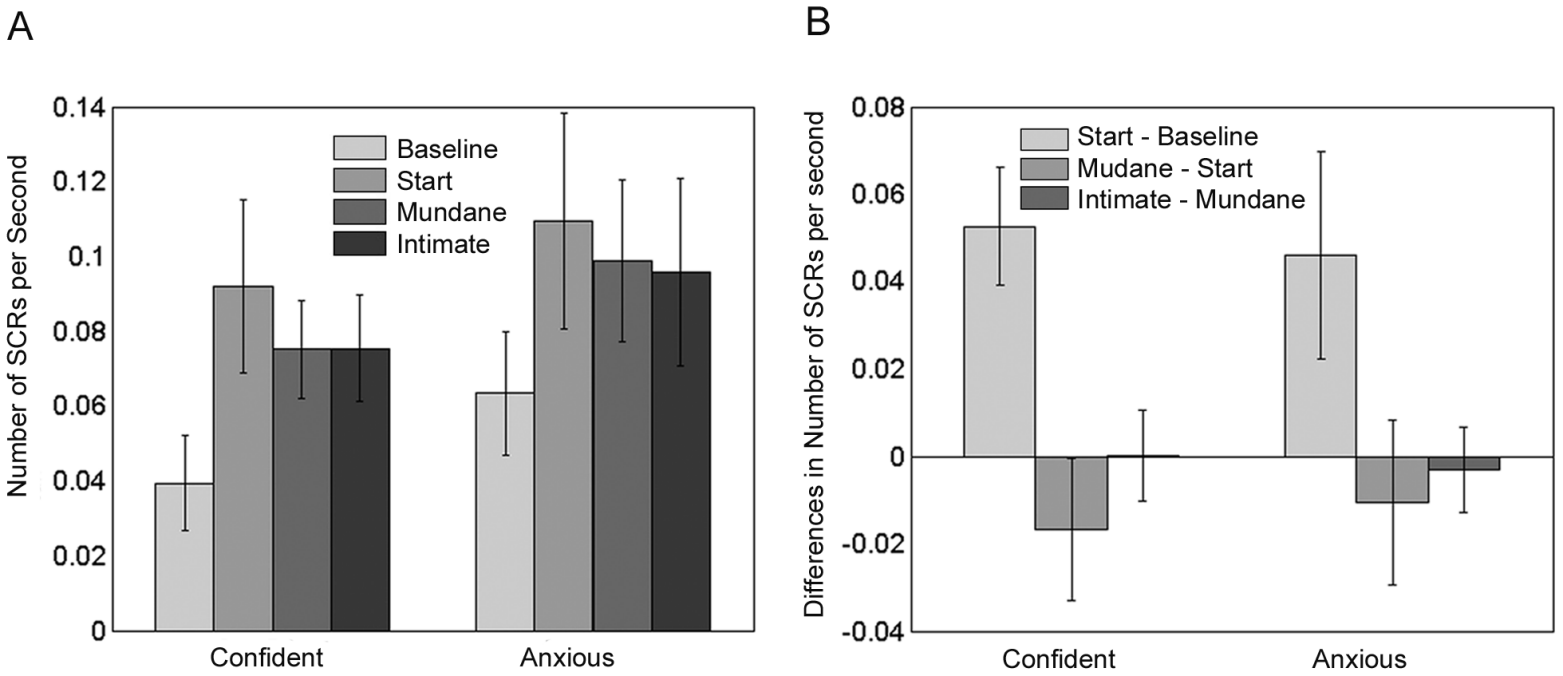
Bar Charts for Number of SCRs per second. (A) Means and Standard Errors of the Number of SCRs per second for the Confident and Anxious groups over the 4 time periods. (B) Means and Standard Errors of the SCR rate differences between successive time periods for the Confident and Anxious groups.

Here we consider the rate and amplitude of skin conductance responses that followed the time periods (Baseline, Start, Mundane, Intimate) as defined in Table 2. The sample size is reduced in this case due to some bad skin conductance readings: n=13 for the confident group and n=14 for the anxious group. Figure 2A shows the result for the number of skin conductance responses per second by group and time period. It can be seen that in both groups there was a significant increase from the baseline to the start period, and then the rate of SCRs remained unchanged. There is no significant difference between the groups (P=0.68). Overall, the difference between the Baseline and Start period is significant (P<0.002). A square root transformation was used to obtain normally distributed residuals, and under this transformation the residual errors are compatible with normality using a Jarque-Bera test (P>0.5). There are no significant differences between the time periods or the groups for the amplitudes.

Figure 2B shows the means of successive differences of the number of SCRs per second. It is clear that there was a large increase in the starting period and then a decline. ANOVA shows a significant difference between the periods, but analysis of the residual errors detected an outlier belonging to the anxious group. On eliminating the outlier the significance level for the difference between the periods is P=0.006, but there is no difference between the Confident and Anxious groups.

### Electrocardiogram

**Figure 3:**
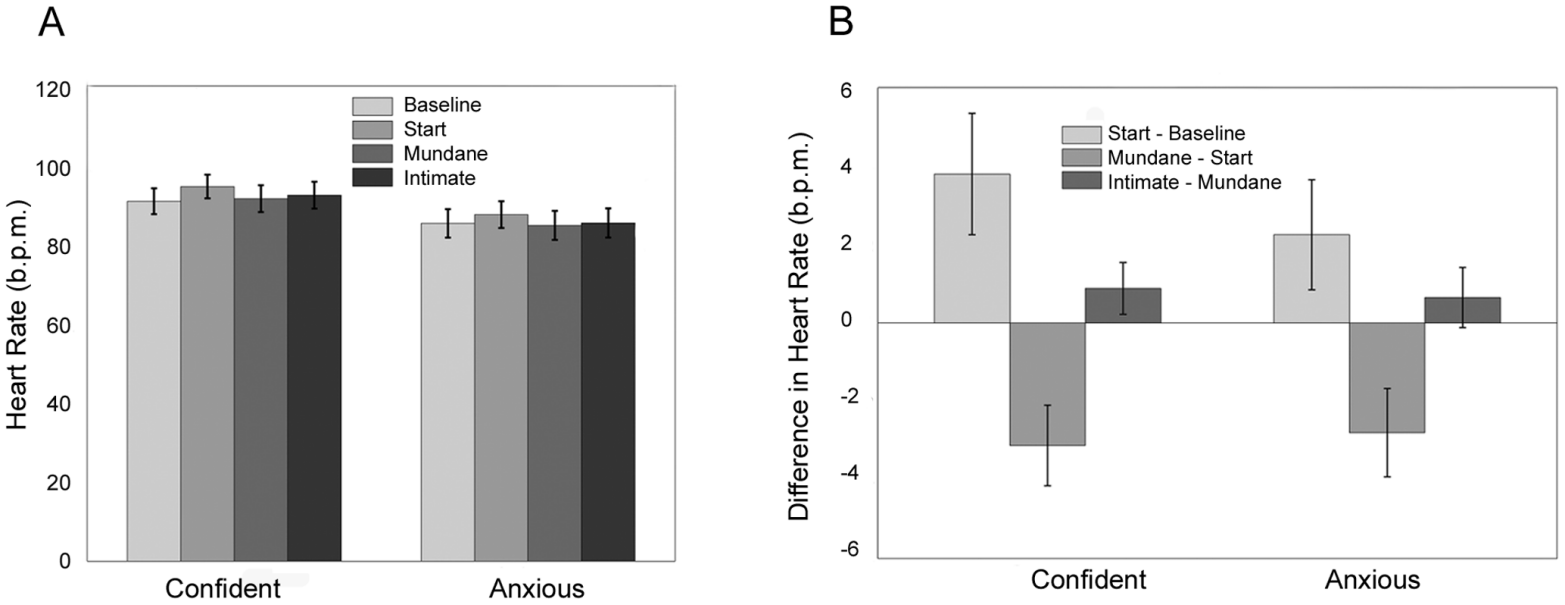
Bar Charts for Heart Rate. (A) Means and Standard Errors of the Heart Rate for the Confident and Anxious groups over the 4 time periods. (B) Means and Standard Errors of Heart Rate Differences between successive time periods for the Confident and Anxious groups.

Figure 3A shows the mean heart rates of the two groups across the 4 time periods. Within groups analysis of variance with a log transform shows a significant difference between the times (P=0.005) but not between the groups (P=0.17), and the residual errors are compatible with a normal distribution using the Jarque-Bera test (P=0.15). A multiple comparisons test (with overall significance at 5%) shows that the Start period has a higher mean heart rate than each of the other three periods.

Figure 3B shows the mean differences in heart rate between the successive periods. The ANOVA shows no differences between the two groups (P=0.54) but a significant difference between the successive time differences (P<0.0000). The residual errors are compatible with normality (P=0.22). A multiple comparisons test shows that Start-Baseline>Mundane-Start, and that Mundane-Start is significantly less than the other two differences (all at the overall 5% level).

**Figure 4:**
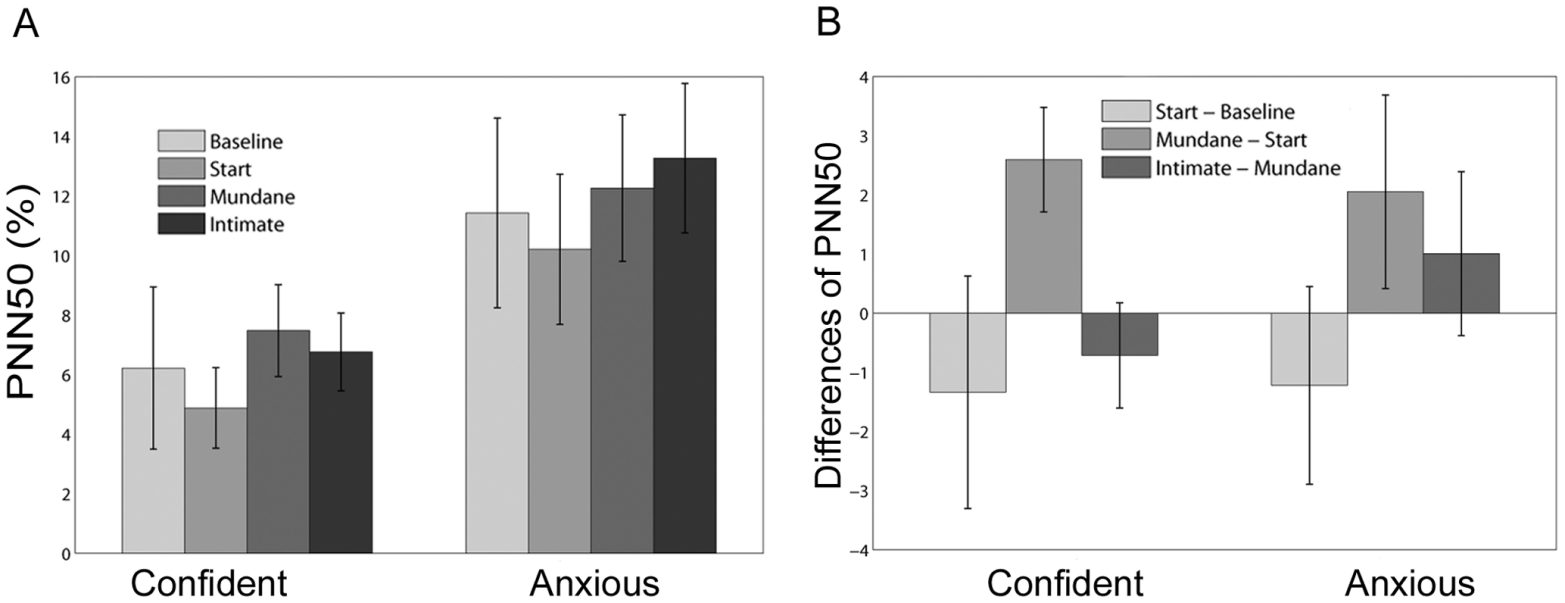
Bar Charts for PNN50. (A) Means and Standard Errors of the PNN50 for the Confident and Anxious groups over the 4 time periods. (B) Means and Standard Errors of PNN50 differences between successive time periods for the Confident and Anxious groups.

Figure 4A shows the mean PNN50 levels across the four periods and for the two groups. The difference between the groups is not significant (P=0.12) but the difference between the means of the periods is significant (P=0.0075). A square root transformation was applied to avoid the problem of non-normal residuals, and under this transformation the residuals are compatible with normality (P>0.5). Multiple comparison tests (P=0.05) show that the mean PNN50 for the Start period is significantly lower than for the Mundane and Intimate periods. Figure 4B shows the differences between the PNN50 values in the successive periods. ANOVA shows that the difference between the periods has significance of P=0.06, but there is no significant difference between the groups. However, the residual errors are not compatible with normality (P=0.02, Jarque-Bera). The family of Box-Cox transformations was tried [42], and one was found that improved the significance for the Jarque-Bera to P=0.04, however, no transformation could be found that fully eliminated the problem of non-normal residuals. However, the distribution of residual errors is close to symmetric with no obvious outliers. When the Box-Cox transformation was used the significance level for the difference between the periods remained at about the same level. Multiple comparison tests show that Start – Baseline is significantly less than Mundane – Start (at the overall 10% level).

### The Effect of Being Single

The Christina avatar asked participants during the experiment whether they had a partner or were single, and the answer was confirmed in the subsequent interview. 8/18 in the Confident group compared to 13/18 in the Anxious group reported that they were single. Considering as before the difference between the post-Exposure and pre-Exposure questionnaires the only significant difference in medians is amongst the single group. Those who are single but Confident have a median difference of 0.36 (IQR: 1.01) while those who are single but Anxious have a median difference of −1.12 (IQR: 1.19). These medians are significantly different from one another (P<0.0045, Mann Whitney U test, two-tailed).

With respect to skin conductance, for those in the partnered group there is no difference between the mean number of skin conductance responses in the successive periods, whereas for those in the single group the difference is significant (P=0.004), with the Start period having a higher mean than the other periods.

The relationships between mean heart rates over the time periods are not different for partnered and single groups compared to the earlier analysis. However, with respect to PNN50, for the partnered group there is a significant difference over the time periods (P=0.036) with the lowest PNN50 occurring during the Start period and the highest level during the Mundane and Intimate periods. However, for the single group there is no significant change of PNN50 over the time periods (P=0.308). It is interesting point that the PNN50 dropped for the partnered group during the initial approach by the woman. It could be that the partnered group became more stressed precisely because in real life they had a partner.

Those who were single had lower average post-Experience questionnaire score compared to the pre-Experience score, and an increase in arousal as measured by the mean number of skin conductance responses during the Start period. Whereas for the partnered group there was a change in mean level of PNN50 over time, there was no change with respect to the single group. These results should be treated with caution, since ‘being single’ was not part of the experimental design but the analysis was based on information obtained during the experiment.

In the post experimental interviews that were conducted (see the next section) the issue of being single arose in the discussion with two of the participants. With one the interviewer had asked about his emotional responses during the conversation with the virtual woman. He answered: “Like the question, ‘are you single?’ I really responded as if she were real. I said, oh, sorry I'm married. Like really to react, I think that was full of emotion. Like someone's courting me, want to be with me of course, but I'm married."

With another participant (P), there was a dialogue with the interviewer (I) as follows:

I: You said [to the virtual woman that] you are single, but you paused a long time, can I ask if you lied?

P: No, I am single.

I: But why did you pause for a couple of seconds?

P: Because I only recently became single.

I: You don't need to be honest to her, she's not real.

P: Exactly. Exactly. It [was the] kind of apprehension that probably if anybody asked that question. Because it was reality I suppose, the fact that it's similar to be if anybody asked me.

### Presence: Response as if Real

The physiological responses suggest that on the average participants had a greater stress level as the encounter with the virtual woman started which then reduced during the period of discussion about mundane matters, and never really picked up again. However, how much were people taking the situation as if it were real, and responding appropriately? In a post experience questionnaire a number of questions addressed this issue, shown in Table 3. There were no differences between the responses given by the Confident and Anxious groups, as expected. (Mann-Whitney U tests on the differences in medians between the two groups had the smallest significance level at 0.41). Table 3 shows the significance levels for sign tests of the hypotheses that the median responses were 4 (the half way mark on the questionnaires scale). We can conclude that on the average participants reported that they behaved as if they were at a party, said what they might have normally said in such a situation, found themselves to be behaving as if it were real in spite of knowing for sure that it was not, and with personal estimates of their own physiological responses appropriate to the situation.

With regard to their encounter with the virtual woman they reported that they were automatically behaving as if she were real in spite of knowing that she was not, and saying things to her that they might have said in a real situation. However, they reported that their emotional response was not similar to how it would have been in a real situation, and nor were their thoughts.

It should also be noted that those in the Anxious group reported somewhat greater anxiety on the initial approach of the woman. In answer to the question “To what extent, if at all, did you become anxious when the woman approached you and started talking with you?" the median responses were 2 and 3.5 for the Confident and Anxious groups respectively (P=0.06, Mann Whitney U test, two-sided).

An interesting observation is that during the conversation people would often offer much more information than was necessary in answer to a question. For example, consider the following dialogue:

C: I'm an air hostess.

P: Really? [sounding impressed]

C: I just arrived in London yesterday, where do you live?

P: I live in [deleted for anonymity]

C: Is that a nice area?

P: It's nice enough I suppose, it's near the Heath. Where have you moved to?

Why did the participant ask about where she had moved to? Even more – why mention that “it's near the Heath?", this information is not called for. There were many examples of this where participants would give answers to questions that were beyond the call of what was needed simply to follow along the conversation. This is compelling evidence that many participants could not help but to become involved in the conversation, treating it as a normal one, in spite of their sure knowledge that this was not a real person. Recall also that the experimenter was out of sight during the experiment, and as far as the participants knew, was unable to hear the conversation. Examples of this type of interaction, suggesting that participants treated the conversation as real can be found in Video S1.

A sample of the participants were interviewed immediately after the experiment. We report a selection of unedited typical comments that gives some insight into the responses of the participants, both positive and negative towards their experience (Text S3). This focuses mainly on their responses to one question: “How much was your behaviour like being in a party?"

## Discussion

In spite of the fact that the virtual environment and the depiction of the virtual characters did not look realistic, participants nevertheless responded quite realistically, as evidenced from the physiological responses, the questionnaires, and interview data. Another way to put this is that the virtual character Christina had some social influence on the participants. In order to explain this we can turn to the model of social influence in virtual environments proposed in [43]. This model suggests that the degree of social influence is a function of two main factors: behavioural realism and social presence. Behavioural realism refers to the realism of the behaviour of the virtual characters with whom the participant interacts (it could also refer to the behaviours of other types of object, but here we are only interested in humanoid character behaviour). Social presence refers to the degree to which participants believe that they are in the presence of representations of other humans. The model suggests that behavioural realism and social presence act together such that when a threshold is met there will be a greater chance of social influence of the virtual characters on the human participant. In particular it is suggested that a high value of one of these factors can compensate for a low value of one of the others.

Now our study was not designed to test this model, but it is useful to consider the outcome in its terms. We would argue that the behavioural realism of the Christina avatar was high relative to its appearance. It gestured as it spoke, looked towards the participant appropriately (e.g., looking down towards his trousers when mentioning them) and moved closer when beginning to talk about more intimate matters. Moreover, although its visual appearance was somewhat cartoonish, the voice was real, and the conversation between participants and the character sound like normal conversations (Video S1). Regarding social presence, the questionnaire data suggests that participants tended to respond realistically to the virtual woman as noted above. Both of these factors together contributed to the likelihood that participants would be influenced by the virtual character.

However, the model also postulates two moderating factors. One is the extent of the relevance of the situation to the participant. For example, an interaction between the participant and virtual character that had little social meaning for the participant could lower the overall threshold for the two main factors – for example, requiring less behavioural realism than when the interaction is important. In our case the interaction would certainly have been important for all participants – being about relationships. In fact the group for whom it would have been most important would be the single participants, since being single the question of relationships may play a larger role in their life. It is precisely in this group where the greatest effects were found. The other moderating factor refers to the target response system – whether the influence is on relatively low level systems such as physiological responses or high level such as emotional or cognitive responses. It should be easier to influence a low level system with relatively crude interaction, whereas more complex responses require more complex behavioural realism and social presence. Again this acts as a way to shift the overall threshold, higher in the case of more complex responses. In our study we have considered a range of responses from physiological through to subjective questionnaire based responses. Regarding the latter recall that participants did tend to report that while they had automatic responses towards the Christina avatar as if she were real, they did not have realistic emotional and cognitive responses. This also fits with the paradigm. Overall then, this model of social influence provides a useful way to frame the overall experimental situation and results, and can be a guide to future studies.

There are three main conclusions from this study. The first is that those in the more anxious group showed a decrease in their post exposure anxiety score compared to their pre exposure score, meaning that their response to the specific event carried with it less anxiety than would be predicted from their everyday life experience. This did not occur for the more confident group. Moreover, this effect was most pronounced for those who were single – being in the anxious group and being single accounted for the greatest fall in score. Second, on average there were no differences in physiological responses between the Confident and Anxious groups, although again this may have been modulated by whether the participants were single or not – for example, being single accounted for the increase in arousal at the start of the encounter with the virtual woman. Third, in the initial period of the experiment when the virtual woman first approached them, participants had an increase in stress, indicated by greater heart rate and lower heart rate variability. This stress reduced during the subsequent period of conversation about mundane matters, and after that the stress never returned at a statistically significant level.

The factor that controlled whether other virtual characters in the scene looked towards the participant or not had no significant influence on the physiological results. However, those in the Anxious group did report a greater sense of being disturbed if the other characters looked towards them, which is consistent with what would be expected for social anxiety [32].

It should be noted that the participants in the Anxious group were moderately socially anxious and did not suffer from extreme social anxiety (this is clear from the scores to the pre-Exposure questionnaire and the interviews) and some of them did have partners. Another point to take into account is that the main experimenter was a woman, which also could have played a role in the outcomes – perhaps even her own responses to the more shy males were unconsciously different to the more confident males. This type of problem is very hard to overcome, since the experiment has to be done by someone – and there would be problems whether carried out by a male or by a female experimenter.

One way in which future studies will be improved is by taking account of recent advances in the understanding of presence. Presence, the tendency of people to respond realistically to situations and events with IVR, has been argued to have at least two independent components, Place Illusion (PI), and Plausibility (Psi) [44], [45]. PI is the illusion of being in the place depicted in the IVR, and this is thought to be largely a function of the sensorimotor contingencies for perception that are afforded by the system. The extent to which people can use their whole bodies to perceive, much as in physical reality, by head turns, body turns, reaching, bending, and so on, is determined by the tracking and display characteristics of the system. Visual sensorimotor contingencies are supported in the Cave through head-tracking and the wide field of view and relatively high resolution display. However, the sound was not spatialised, which is an important point for improvement in subsequent work, especially in scenarios that will involve multiple virtual characters.

Psi is the illusion that what is happening is really happening (notwithstanding the certain knowledge that it is only a simulation). This is thought to depend on three factors the extent to which (a) events occur in the IVR that correlate with the actions of the participant (b) events relate personally to the participant, and (c) the events and situation are credible with respect to a similar real-life situation.

Condition (a) was fulfilled in this experiment through the seemingly interactive nature of the conversation. The simplest example of this is that when the participant asked the character to repeat a phrase, she did so, and in some cases there were several different recordings of the same phrase in order to make this possible in a way that hid the fact that the responses were pre-recorded. Another type of example involved phrases that seemed to indicate that the avatar had been listening to the previous statements of the participant. For example, one section of the dialogue was:

C: So, what are you doing for a living?

P: Currently a student at xxx.

C: Oh, what's your subject?

P: I study xxx.

C: Very interesting, tell me more.

Another example suggested that she was visually aware of the participant:

C: I've noticed that people dress very well around here. By the way, that shirt looks great on you. How much was it?

P: Ah, thank you. It was 15 pounds.

C: Ah, I really want to find a pair of trousers, something like these [Looking down] for my brother. Where did you get these?

P: I've got them in New York in the US.

However, as mentioned by some of the participants in the interviews, this also requires improvement, with the possibility of more variety in the range of answers given by the avatar, and also faster response times. In this experiment the avatar's responses to unforeseen interventions by the participant were chosen by the operator from an array of possible responses on screen, thus there was inevitably a delay in finding the right response in some circumstances.

Regarding point (b) this was clearly fulfilled in this experiment since the whole experience was directed personally to the participant. However (c) was more problematic, obtaining a scenario that appears to conform to expectations from everyday reality, but nevertheless going beyond this in order to achieve a therapeutic effect. The very fact of going alone to the party (for some without the safety valve of alcohol) was already problematic. It would not be difficult to design a future scenario where the participant was accompanied either by another actual person (a confederate) or a virtual person. Some alcohol could be available, subject to ethical evaluation. However, it would have to be considered whether the inclusion of such safety behaviours militates against the possibility of therapeutic gain, since such safety features are designed by the participants precisely to avoid the anxiety provoking situation. Of course it is always possible in a series of trials to include safety features in the earlier trials and then remove them in later ones.

Second, there is the issue of various aspects of realism. We have found in other work that illumination realism does contribute to Psi [45], which is something now relatively straightforward to achieve. It is obvious that the graphical appearance of the avatars and their facial expressions need to be improved. This has already been accomplished in more recent work, and is no longer much of a technical problem. Providing appropriate smell for such a scenario is likely to also be a powerful enhancement to realism, although more difficult to achieve technically. The feeling of touch from the avatar would also be likely to contribute significantly to Psi – for example, she would reach out and touch the arm of the participant who would synchronously feel the touch. This was achieved in [46], though would be more complex to implement in a Cave VR system, since it would be critical that the participant should not see the haptic devices. However, a haptic jacket that the participant wears rather than external force-feedback devices may work well.

Turning now to methodological issues it could be argued that the change in post-exposure questionnaire score for the Anxious group is due to regression to the mean. Since in the selection process we chose the lowest and highest scorers from the pre-Exposure questionnaire there could be regression to the mean in the scores of the post-Exposure questionnaire. However, based on the same reasoning it would be expected that the average score of the Confident group should increase, which did not happen.

A further methodological issue is that we used alternate forms of the SPAI questionnaire for the pre and post measurements. It could be argued that this introduced a source of non-comparability. However, the two forms differed only minimally, and we present data in the supplementary material to support their equivalence. This is an important issue and it is worth reiterating our methodology since it may be regarded as a weakness of the study that identical pre- and post-test questionnaires were not used.

Using identical pre- and post-test questionnaires is, of course, critical in situations where a *treatment* has been given to a patient group. The purpose of the post-test is precisely to examine whether the treatment has had an effect in comparison to the pre-test. However, we have not carried out a treatment, but rather an *experiment* to examine the differential effects on a group more confident in their social relationships with members of the opposite sex and another more anxious group. We used a subset of the SPAI questionnaire as a source of questions for sample selection, and then the post questionnaire as an alternate form of the SPAI. We have shown that with respect to the Confident group that the post-Experience questionnaire is not only highly correlated with the pre-Experience questionnaire but that the regression line is at 45 degrees through the origin. However, this is not the case for the Anxious group, so something different happened for this group. Our interpretation, supported by the data, is that their post-Exposure score, in this specific circumstance, was less than would have been expected in comparison to their pre-Exposure questionnaire scores.

In fact prior to the study we had expected that those in the Anxious group would have their anxiety dramatically *increased* by this encounter – recall that we specifically selected on questions concerned with difficulties in interacting with members of the opposite sex. The physiological evidence suggests that both Confident and Anxious groups experienced an initial increase in anxiety. However, the greatest change in questionnaire scores from the encounter was for those in the Anxious group. Their post-Exposure scores were lower than would have been predicted from their pre-Exposure questionnaire scores with respect to how they feel in everyday life. This is the most promising and interesting result of this study. We speculate that the mundane period was what was important in relaxing the participants. After the surprise and shock of the initial encounter, the conversation turned to every day non-threatening matters, about living in London and the weather, before leading on to the more intimate topics. Another way to look at this is that the Anxious group may have had greater anticipatory anxiety from the moment that they entered the party and especially as the virtual woman approached them. The Mundane period showed that their worst fears were not going to be realised, and their post questionnaire ratings reflected their relief that the encounter had been more pleasant than they initially feared.

If VR is to be used in the treatment of social anxiety then the experiment described in this paper shows a particular strategy that could be adopted. A major problem with people who have the kind of social anxiety considered in this paper is initiation – they find it difficult to initiate an interaction with a member of the opposite sex, and engage in various safety behaviours [32] to try to overcome this problem, such as alcohol and always making sure that they are with friends whenever they engage in a new social encounter such as attending a party – as reported by some of the participants in their interviews. However, in virtual reality the problem of the initial encounter is easily solved. Since the probability of the events that occurred in our experiment occurring in a real social gathering is very low, and given the problem of initiating an encounter, it is likely that a major problem with socially anxious males is their lack of experience. They cannot approach a woman, and social convention makes it unlikely that they will be approached, and in any case they would avoid situations where this would happen (since they would not in any case go alone to such a social encounter). Just as in the case of fear of heights a person with acrophobia would not go to a high place in physical reality but would in virtual reality, so the same may be true in the social situation.

Now although the problem of initiation can be solved in virtual reality, inevitably the participant is likely to experience heightened stress in the initial encounter. But since the ‘work’ is being done by the virtual character, she being the one to push the conversation along, this is stress is manageable. If then the conversation turns to every-day mundane matters, not touching at all on the question of personal relationships, our evidence suggests that this reduces the stress of the participants, which does not rise again even though the conversation turns to more intimate matters. We suggest that a strategy that employs interspersing mundane conversation with conversation that is potentially more stressing, could result in the building up of confidence over time, and give participants experience and training in such social encounters.

In our next study we will concentrate on people with strong social anxiety issues, and employ the kind of virtual reality strategy outlined above, interspersing mundane matters with more intimate stretches of conversation. In particular we would test the specific hypothesis that interspersing mundane conversation with more intimate conversation would lead to a faster reduction in anxiety than strategies that maintained a strong level of intimate conversation throughout. Additionally the experiment was a few minutes, but in reality such people may need many such reinforcing encounters, and meet a variety of types of virtual character with different types of behaviour – more or less aggressive, more or less shy in their own approach, finally terminating in a series of trials where the participant is given opportunities to make the first approach himself.

This was a complex experiment with many features that have not been attempted before – a sustained dialogue between a person and a virtual character in an atypical social situation. It was encouraging to note that in spite of the relative increase in physiologically measured anxiety at the initial approach of the virtual woman, that the more socially anxious participants appeared to become less stressed over time. The idea of taking the pressure of initiating social contact away from socially anxious individuals, and the use of mundane confidence-building conversation provide interesting ways forward in the design of programs to help people who suffer from this type of often debilitating anxiety.

## Supporting Information

Table S1 **The Pre- and Post-Exposure Questionnaires in Relation to the SPAI.**
(PDF)

Text S1 **An example of a complete dialog.**
(PDF)

Text S2 **Correlation Coefficient Between the Sum of a Set of Random Variables, and the Sum of a Subset of that Set.**
(PDF)

Text S3 **A Selection of Responses to the Interview Question “How much was your behaviour like being in a party?"**
(PDF)

Video S1 **A video of one complete experiment after the baseline period.**
(WMV)
