# Supplementary material for: Socially Anxious and Confident Men Interact with a Forward Virtual Woman: An Experimental Study
Source: PLoS One. 2012 Apr 11;7(4):e32931. doi: 10.1371/journal.pone.0032931 (PMC3324473; doi:10.1371/journal.pone.0032931)
Supplement: Table S1 — The Pre- and Post-Exposure Questionnaires in Relation to the SPAI. (PDF) [file pone.0032931.s001.pdf]

Socially Anxious and Confident Men Interact with a Forward Virtual Woman: An Experiment Study

Xueni Pan, Marco Gillies, Chris Barker, David Clark, Mel Slater

Supporting Table S1

The Pre- and Post-Exposure Questionnaires in Relation to the SPAI

The first column shows the number of the item in the full SPAI questionnaire. Where an item has more than one question these are labelled as a,b,c,…. The second column shows the correspondence between the pre-Exposure Questionnaire and the SPAI. For example, Q1 in the pre-Exposure is equal to question 9a in the full SPAI. The third column shows the post-Exposure questions. For example, Q2 in the post-Exposure is based on Q3 in the full SPAI. Some of the questions are given but not all since the SPAI is a commercial product.

Full SPAI	Pre-Exposure	Post-Exposure
1		1
3. I feel anxious when I am in a social situation and I become the center of attention.		2. I felt anxious when I thought I might have become the centre of attention.
4		3
7		4
8		5
9a. I feel anxious when in small gatherings with strangers.	1	6. I felt anxious because there were strangers.
9b. I feel anxious when in small gatherings with authority figures.	2	
9c. I feel anxious when in small gatherings with opposite sex.	3	7. I felt anxious because there were members of the opposite sex.
9d. I feel anxious when in small gatherings with people in general.	4	8. I felt anxious because there were the people there in general.
11a	5	
11b	6	
11c	7	
11d	8	
12a. I feel anxious and I do not	9	9. I felt anxious and did not

know what to do when in a new situation with strangers.		know what to do in this new situation with strangers.
12b	10	
12c. I feel anxious and I do not know what to do when in a new situation with opposite sex.	11	10. I felt anxious and did not know what to do in this new situation with members of the opposite sex.
12d. I feel anxious and I do not know what to do when in a new situation with people in general.	12	11 I felt anxious and did not know what to do in this new situation with the people there in general.
15a	13	
15b	14	
15c	15	
15d	16	
18a. I feel anxious when approaching and/or initiating a conversation with strangers.	17	12. I felt anxious about approaching or initiating a conversation with strangers.
18b	18	
18c. I feel anxious when approaching and/or initiating a conversation with opposite sex.	19	13. I felt anxious about approaching or initiating a conversation members of the opposite sex.
18d. I feel anxious when approaching and/or initiating a conversation with people in general.	20	14. I felt anxious about approaching or initiating a conversation with the people there in general.
19a. I feel anxious when having to interact longer than a few minutes with strangers.	21	15. I felt anxious because I had to interact for a while with strangers.
19b	22	
19c. I feel anxious when having to interact longer than a few minutes with opposite sex.	23	16. I felt anxious because I had to interact for a while with members of the opposite sex.
19d. I feel anxious when having to interact longer than a few minutes with people in general.	24	17. I felt anxious because I had to interact for a while with....The people there in general.
23a	25	
23b	26	
23c	27	
23d	28	
24a	29	
24b	30	

24c	31	
24d	32	
25a		18
25c		19
25d. I leave social situations where there are people in general.		20. I wanted to leave the party because there were the people there in general.
26a		21
26b		22
26c. Before entering a social situation, I think about all the thing that can go wrong. The types of thoughts I experience are: What will I do if no one speaks to me?		23. While I was in the party I was thinking about the things that might be going wrong in that situation. The types of thoughts I experienced were: What if no one speaks to me?
26d		24
28		25
29		26
30a		27
30b		28
30c		29
30d. I experience troublesome thoughts when I am in a social setting. For example: whatever I will say it will probably sound stupid.		30. I experienced troublesome thoughts. For example: whatever I will say it will probably sound stupid.
32a		31
32b		32
32c		33
32d		34
32e		35
Own Questions:	I feel anxious when being approached by…	
	33. strangers	
	34. authority figures	
	35. opposite sex	
	36. people in general.	
