# Supplementary material for: Socially Anxious and Confident Men Interact with a Forward Virtual Woman: An Experimental Study
Source: PLoS One. 2012 Apr 11;7(4):e32931. doi: 10.1371/journal.pone.0032931 (PMC3324473; doi:10.1371/journal.pone.0032931)
Supplement: Text S1 — An example of a complete dialog. (PDF) [file pone.0032931.s002.pdf]

Socially Anxious and Confident Men Interact with a Forward Virtual Woman: An Experiment Study

Xueni Pan, Marco Gillies, Chris Barker, David Clark, Mel Slater

Supporting Text S1

An example of a complete dialog

Christina starts looking at the participant, and then makes her way towards him.
[C: Christina; P: Participant]

C: Hi, It looks like we are the only people alone here, right?
P: Ah yes.
C: My name is Christina. What's your name?
P: My name is xxx.
C: It's very nice to meet you.
P: Nice to meet you.
C: So, what are you doing for a living?
P: Current a student at xxx.
C: Oh, what's your subject?
P: I study xxx.
C: Very interesting, tell me more.
P: Basically we study xxx
C: enhen.
P: And I'm interested in xxx.
C: I'm an air hostess; I just arrived in London yesterday. Where do you live?
P: Sorry?
C: Where do you live?
P: Ah I live in xxx.
C: It's that a nice area?
P: I'd like to think so, but no, it's a bit rough.
C: I don't know London very well, but actually, I am thinking about moving here, what do you think?
P: Yeah definitely, London is great!
C: But I heard it rains all the time here, is that true?
P: En, no, not all the time. Once a week maybe?
C: Well, the weather is not that important to me. Have you lived here long?
P: Yeah, I've lived here all my life.
C: Do you like it here?
P: Err, it's too busy…too many people.
C: I've noticed that people dress very well around here. By the way, that shirt looks great on you. How much was it?

P: Ah, thank you. It was 15 pounds.
C: Ah, I really want to find a pair of trousers, something like those [Looking down] for my brother. Where did you get these?
P: I got them in New York in the US.
C: So, do you know anyone here?
P: Just you.
C: I feel a bit shy about talking with other people, do you mind if I talk with you for a bit longer? [start moving towards the participant to an intimate distance].
P: Don't worry about it. It's OK.
C: If you don't mind me saying, I think you look very nice.
P: Hahaha, you look very well yourself.
C: I was wondering actually, are you single, or involved with someone at this time?
P: I'm involved with someone.
C: Actually, it is the same with me.
P: OK.
C: Anyway, what are you doing after this party?
P: Probably going out with friends. How about you?
C: Alright, I see. Maybe we should meet up.
P: Yes, sure.
C: I just need to go out for a moment, shall we meet by the entrance of this building later?
P: Sounds good.
C: See you later!
P: Bye!
