# Supplementary material for: Socially Anxious and Confident Men Interact with a Forward Virtual Woman: An Experimental Study
Source: PLoS One. 2012 Apr 11;7(4):e32931. doi: 10.1371/journal.pone.0032931 (PMC3324473; doi:10.1371/journal.pone.0032931)
Supplement: Text S2 — Correlation Coefficient Between the Sum of a Set of Random Variables, and the Sum of a Subset of that Set. (PDF) [file pone.0032931.s003.pdf]

Socially Anxious and Confident Men Interact with a Forward Virtual Woman: An Experiment Study

Xueni Pan, Marco Gillies, Chris Barker, David Clark, Mel Slater

Supporting Text S2

Correlation Coefficient Between the Sum of a Set of Random Variables, and the Sum of a Subset of that Set

The motivation of this section is to show that there is likely to be a high positive correlation between the sum of scores of a subset of a questionnaire and the sum of scores of the questions in a full questionnaire.

In general let $x_1,x_2,...,x_n$ be a set of random variables each with mean 0 and variance 1. Let $\rho_{ij} = E(x_i x_j)$ be the covariance between x_i and x_j, which is the same as the correlation under the unit variance assumption.

Let $s_k = \sum_{i=1}^{k} x_j$ with s_n being the full sum and $k \leq n$. We find the correlation $\rho(k,n)$ between s_k and s_n:

EQ1:

$$\rho(k,n) = \frac{\mathrm{cov}(s_k,s_n)}{\sqrt{\mathrm{var}(s_k)\,\mathrm{var}(s_n)}}$$

EQ2:

$$\begin{aligned}
\mathrm{cov}(s_k,s_n) &= E\left(\sum_{i=1}^{k} x_i \sum_{j=1}^{n} x_j\right) \\
&= E\left(\sum_{i=1}^{k} x_i^2 + 2\sum_{i=1}^{k-1}\sum_{j=i+1}^{k} x_i x_j + 2\sum_{i=1}^{k}\sum_{j=k+1}^{n} x_i x_j\right) \\
&= k + 2\sum_{i=1}^{k-1}\sum_{j=i+1}^{k} \rho_{ij} + \sum_{i=1}^{k}\sum_{j=k+1}^{n} \rho_{ij}
\end{aligned}$$

Similarly,

EQ3:

$$\begin{aligned}
\mathrm{var}(s_k) &= E\left[\left(\sum_{i=1}^{k} x_i\right)^2\right] \\
&= k + 2\sum_{i=1}^{k-1}\sum_{j=i+1}^{k} \rho_{ij}
\end{aligned}$$

Therefore from EQs 1-3:

EQ4:

$$\rho(k,n) = \frac{k + 2\sum_{i=1}^{k-1}\sum_{j=i+1}^{k}\rho_{ij} + \sum_{i=1}^{k}\sum_{j=k+1}^{n}\rho_{ij}}{\sqrt{\left(k + 2\sum_{i=1}^{k-1}\sum_{j=i+1}^{k}\rho_{ij}\right)\left(n + 2\sum_{i=1}^{n-1}\sum_{j=i+1}^{n}\rho_{ij}\right)}}.$$

Suppose that $\rho_0 \le \rho_{ij} \le \rho_1$. Then

EQ5:

$$\rho(k,n) \ge \sqrt{\frac{k}{n}}\left(\frac{1 + (n-1)\rho_0}{\sqrt{(1 + (k-1)\rho_1)(1 + (n-1)\rho_1)}}\right).$$

In the situation where ρ_0 and ρ_1 are close we can replace the correlations by $\bar{\rho}$ (say the mean of all the correlations), in which case EQ4 becomes:

EQ6:

$$\bar{\rho}(k,n) \approx \sqrt{\left(\frac{k}{n}\right)\left(\frac{1 + (n-1)\bar{\rho}}{1 + (k-1)\bar{\rho}}\right)}.$$

To get more insight into the meaning of EQ5, we suppose that ρ_1 will be close to its upper bound of 1. Then the approximate lower bound becomes

EQ7:

$$\begin{aligned}\rho(k,n) &\ge\approx \frac{1}{n} + \left(\frac{n-1}{n}\right)\rho_0 \\ &\approx \rho_0\end{aligned}$$

since n would typically be large (e.g., 97 different questions in the social phobia part of the SPAI).

Now we interpret the x_i as being scores on individual questions in a questionnaire. In the SPAI many questions are grouped into items and we have not found a data source that provides the correlations between individual questions. However, we do have the 153 completed questionnaires from the recruitment process. These answered 32

questions from the SPAI and one additional set of questions. If we consider the responses to the 32 SPAI questions we have:

$$\rho_0 = 0.3421$$
$$\rho_1 = 0.8833$$
$$\bar{\rho} = 0.5769.$$

Using EQ5

$$\rho(k,n) \geq 0.3431$$
$$\bar{\rho}(k,n) = 0.9925.$$

The minimum bound is already quite high as a theoretical correlation, and mathematically it may not be the greatest lower bound. It provides some evidence that our subsets of the SPAI questionnaire should have strong positive correlation with what might have been obtained with the full SPAI.

See also [1] (Chapter 8) discussing the Spearman-Brown formula, an argument closely resembling the one above. Classical testing theory generally assumes equal co-variances between items further justifying our argument.

Reference

1. Gulliksen, H., *Theory of mental tests*. 1987, Hillsdale, New Jersey and London: Lawrence Erlbaum Associates.
