# Supplementary material for: Socially Anxious and Confident Men Interact with a Forward Virtual Woman: An Experimental Study
Source: PLoS One. 2012 Apr 11;7(4):e32931. doi: 10.1371/journal.pone.0032931 (PMC3324473; doi:10.1371/journal.pone.0032931)
Supplement: Text S3 — A Selection of Responses to the Interview Question “How much was your behaviour like being in a party?" (PDF) [file pone.0032931.s004.pdf]

Socially Anxious and Confident Men Interact with a Forward Virtual Woman: An Experiment Study

Xueni Pan, Marco Gillies, Chris Barker, David Clark, Mel Slater

Supporting Text S3

A Selection of Responses to the Interview Question “How much was your behaviour like being in a party?”

Anxious Group

I think I was acting as if I was in such a situation, but I think to really grasp how I would act in such situation it needed to be a bit longer.

I think it’s very similar. In the sense that once I found a companion it was easy to be relaxed, so I was able to stay in the party.

I don’t think it was very realistic because I was always kind of conscious that it was a virtual environment. It’s interactions with images rather than people.

Probably not too much…At a normal party I would have had some alcohol before hand….Because that’s what I always do.

Not that much. Because the character was in a cubic shape, they are not so close to real characters, as well as the fact the responses were pre-recorded. And especially when I realise the fact I couldn’t listen to the language very closely, and I ask for repetition, and the system makes some...delay. This delay immediately reminds me that, this is not real situation. …For example towards the end when the woman approached more and made some sexual advances, that will be very unlikely for me to respond the way I did. For example she said let’s meet outside, I wouldn’t be like, all right, let’s go. It’s different from what I would have done in a real life situation. I was kind of surprised of my response, this artificial thing, so what I say doesn’t matter. After all I’m not going with her anywhere.

I would say not very much so…I think the other senses are not there, like smell, and I am not comfortable without a drink.

I felt like she was very real. I kind of felt like the experience of talking to a real person, even though I knew I wasn’t, she feels like it. She doesn’t give much time to think anything else. It was just like, oh dear, what am I going to say now?

Pretty much the same as I would behave in a real situation.

Confident Group

That was really weird….I think it was more psychological. First I was surprised to see, ´cause it was quite static against the wall, and then she sort of walked out towards you. That was weird. Also cause her movements was sort of real, so it's quite weird to see something looks quite real but not real coming towards you.

Not really because it felt more like a networking session than a relaxing party…. I was surprised that I am a little bit shy around people and so I was surprised that I felt, I blush easily, but I was surprised I thought I blushed maybe the same way…. As soon as I realised I was being addressed, that virtual character just talking to me [in the beginning of the conversation] … and then again when she walked closer…. It was comical because of the way she walking. But it was weird. I didn't feel like I've moved, but I felt the urge of getting backwards.

When she started approaching me, I think I was aware of the situation, but I was still responding to it, to the visual aspect. … Well, I cannot say sexual attraction because it wasn't real but it was certain, when her face was coming straight at me it was sort of, I wouldn't know how to define but some kind of arousal, not physical, just pleasurable, you know. [Compared to a real woman] … I will probably be more aggressive. Here she started talking to me, but in real life I would be starting the conversation rather than making only response.

It was almost like real. Because you walked in, you saw all the people, it looks real, you real feel it's normal. Because you see other people, they are moving, they are talking, it's just like a real situation.

It was very real. The graphic could be little bit better. But it didn't matter that much. I sort just get used to it.

Well I thought it was close to the behaviour in normal party, but … you have to give the person a bit more feeling like in a real world like visual audio something. I thought the light was a bit low. Also the volume should have been higher. … I have that thought in mind that she's not real. But I was really engaged into it. So I think acted as a real situation….in a real party people don't approach you that often. Somebody has to take the initiative. I belong to that group who usually take the initiative. … I got the idea from the beginning that it was quite clingy, like people have their own groups, and I thought that was the thing, because she was like hey we are the only ones alone here. This is what usually happens when you go to a bar you usually go with a couple of friends and all that, and meet new people there. But you are not completely alone, it doesn't quite happen. People don't just say hey I'm going alone to a bar.

Yeah, I think it was quite different from a party….I think I was aware she wasn't real. I don't know if you intended to make her not to look real but, the graphic aren't realistic. You can see the frames around the floor. I'm not sure about [whether my behaviour was realistic]. You are obviously monitoring my heart and my, voluntary reactions, I don't know what they are in a normal situations. So, possible, I don't know. I think I probably sort of reacted, but I don't think it's the same way….[My answers were] very different cause I wasn't sure if I was suppose to speak to only her, I was sure if I could ask questions, or if I can direct questions to other people. … So you have lots of background thoughts, which you probably won't have in a real party.

In the beginning I didn't feel it was a real party, but then the woman was approaching to me I thought the reaction was like a real reaction.…. I'd say that I was aware that it wasn't a real situation so your behavior is influenced by that. … [I didn't talk much but] Usually I'm very chatty. But the situation you felt it's not real. So I felt stupid talking more because I think the girl has already the answers made up. She will not interact with my answers. I suppose.

… strange to be driven in that completely unreal world, and not noticing anything of the real world outside. Just completely driven into it. … I think it was really close to how I would have done in a real party…. You always know that is unreal. But still I found that you are really driven into it. … It's just like wherever you look, there is no sign of being in the experiment. Well there is sign of being in an experiment, but you don't feel like it. That's a strange feeling.

Yeah quite a lot actually. There were a few things, one being, right at the beginning, she introduced herself and she said her name. And there was a little pause, a little gap. In normal conversation, if there was a gap, I found myself filling the gap, saying something. And that's what I did, I said, "So, …". I asked her something and she said something else back to me. So I found that interesting. Right at the beginning, I'm actually, I felt obliged to fill the gap with small talk like I would in real life. There were two main other things that made me think afterwards why I really respond to it as if it was real. One time I couldn't hear her, and I did this, I turned my head, and I leaned forward. Cause that's what I normally do, to hear what she's saying with my ear. I did that, it was very odd. There was a few times I've noticed I was reacting as I would in real life. Just very peculiar, ′cause I know it wasn't, but I still did. Strange. … If I was polite, I might have said, she was forward and friendly. If I was not polite, I might have said she was desperate. Hahahaha. She was very flattering; maybe if it was a real party and I had dressed nicely and looked nicely, maybe I would think she was honestly telling me I was nice. But her telling me I was good looking and liked the way I dressed, it was all very flattery, very obviously to me, especially when I'm not well dressed…. Another thing that reminded me it wasn't real was because, I'm clearly not dressing well, and she was asking where to buy the clothes. In fact if I was dressed like this at a real party, I might think she was taking the mickey, making fun of me. "You are dressing so well, where can I buy this" [Ironically].
